# Quantum Dot-Loaded Liposomes to Evaluate the Behavior of Drug Carriers after Oral Administration

**DOI:** 10.1155/2013/848275

**Published:** 2013-07-18

**Authors:** Kohei Tahara, Shiho Fujimoto, Fumihiko Fujii, Yuichi Tozuka, Takashi Jin, Hirofumi Takeuchi

**Affiliations:** ^1^Laboratory of Pharmaceutical Engineering, Gifu Pharmaceutical University, 1-25-4 Daigaku-Nishi, Gifu 501-1196, Japan; ^2^Immunology Frontier Research Center, Osaka University, 3-1 Yamadaoka, Suita, Osaka 565-0871, Japan; ^3^Laboratory of Formulation Design and Pharmaceutical Technology, Osaka University of Pharmaceutical Sciences, 4-20-1 Nasahara, Takatsuki, Osaka 569-1094, Japan; ^4^Quantitative Biology Center, RIKEN, 6-2-3 Furuedai, Suita, Osaka 565-0874, Japan

## Abstract

We have developed submicron-sized liposomes modified with a mucoadhesive polymer to enhance peptide drug absorption after oral administration. Liposomal behavior in the gastrointestinal tract is a critical factor for effective peptide drug delivery. The purpose of this study was to prepare quantum dot- (QD-) loaded submicron-sized liposomes and examine liposomal behavior in the body after oral administration using *in vivo* fluorescence imaging. Two types of CdSe/CdZnS QDs with different surface properties were used: hydrophobic (unmodified) QDs and hydrophilic QDs with glutathione (GSH) surface modifications. QD- and GSH-QD-loaded liposomes were prepared by a thin film hydration method. Transmission electron microscopy revealed that QDs were embedded in the liposomal lipid bilayer. Conversely, GSH-QDs were present in the inner aqueous phase. Some of the GSH-QDs were electrostatically associated with the lipid membrane of stearylamine-bearing cationic liposomes. QD-loaded liposomes were detected in Caco-2 cells after exposure to the liposomes, and these liposomes were not toxic to the Caco-2 cells. Furthermore, we evaluated the *in vivo* bioadhesion and intestinal penetration of orally administered QD-loaded liposomes by observing the intestinal segment using confocal laser scanning microscopy.

## 1. Introduction

Liposomes are a very attractive drug delivery system because they are physically and chemically well-characterized structures that can be delivered through almost all routes of administration and are biocompatible [1–3]. We have developed a submicron-sized (100–200 nm) mucoadhesive liposomal system by modifying the liposome surface with a mucoadhesive polymer such as chitosan to achieve an oral peptide formulation [4, 5]. The effectiveness of polymer-modified liposomes was confirmed by the enhanced and prolonged pharmacological effect of peptide drugs such as insulin, which was orally administered in a polymer-coated liposomal form to rats. Therefore, it is important to characterize the mucoadhesive properties of oral liposomal systems *in vivo* based on liposomal behavior in the body [6]. In a previous study, we examined the mucosal layer of the rat intestine to detect organic dye-labeled liposomes by confocal laser scanning microscopy (CLSM) after administering these particulate systems [4]. 

In *in vivo* experiments, near-infrared (NIR) optical imaging is a powerful tool for real-time observation of the dynamic behavior of liposomes because it is a minimally invasive, nonionizing method that permits sensitive deep tissue imaging [7, 8]. However, traditional NIR dyes have several disadvantages for use as fluorescent probes, such as low solubility in aqueous solution, low quantum yield, and low photostability [9].

Semiconductor nanocrystals known as quantum dots (QDs) are fluorescent nanoparticles with diameters of 1–10 nm [10]. QDs have been extensively investigated as optical probes for various biomedical applications *in vitro* and *in vivo* [11–13]. In this present study, we prepared QD-loaded liposomes to examine liposomal behavior in the body after oral administration. Conventional QDs are very hydrophobic and insoluble in water. Therefore, the QDs could be entrapped in the lipid bilayer and not in the inner aqueous phase. We also attempted to encapsulate hydrophobic QDs with glutathione (GSH) surface modifications in the inner water layers of the liposome [14]. The cellular uptake and cytotoxicity of these QD-loaded liposomes were evaluated using Caco-2 cells. The feasibility of detecting liposomal QDs in the gastrointestinal tract was investigated after oral administration to rats in an *in vivo* experiment.

## 2. Materials and Methods

### 2.1. Preparation of QDs Loaded Liposomes

CdSe/CdZnS QDs and GSH-modified CdSe/CdZnS QDs (GSH-QDs) were prepared and characterized as described previously [14, 15]. QD-loaded liposomes were prepared using a thin film hydration method. Typically, a mixture of l-*α*-distearoylphosphatidylcholine (DSPC; Nippon Oil and Fats, Japan), stearylamine (SA, Tokyo Kasei, Japan), dicetyl phosphate (DCP; Sigma, St. Louis, MO, USA), and cholesterol (Chol, Sigma) were dissolved in chloroform in different molar ratios. Final lipid concentration was 10 mM. The solvent was evaporated to dryness, and the lipid film was further dried under vacuum overnight. Multilamellar vesicles were obtained by hydrating the lipid film using phosphate buffer (66.67 mM, pH 7.4). Small unilamellar vesicles were obtained by probe sonication for 5 min in an ice water bath (Sonifier 250; Branson, MO, USA). The hydrophobic QDs were loaded during the preparation of the thin lipid film, and the liposomes were prepared as aforementioned. For loading, GSH-QDs were mixed in an aqueous hydration medium. Unloaded QDs and GSH-QDs were removed by ultracentrifugation in a sucrose density gradient [16]. The resolved fluorescent bands observed under UV illumination (LAS-3000 UV mini; Fuji Film, Tokyo, Japan) were carefully recovered by manual pipetting and analyzed for QD encapsulation. The particle sizes were determined by dynamic light scattering (DLS; Zetasizer Nano ZS, Malvern, Worcestershire, UK). The zeta potential of liposomes was measured by the laser Doppler method (Zetasizer Nano ZS). Fluorescence spectra were recorded at room temperature using a fluorescence spectrophotometer (Model FP-6600; Jasco, Japan). QD-loaded liposomes were observed by transmission electron microscopy (TEM, JEM-1200EX; JEOL, Tokyo, Japan). 

### 2.2. Cell Association Study by CLSM

Human epithelial colorectal adenocarcinoma cells (Caco-2) were used in the cell study. Caco-2 cells were grown on Lab-Tek II Chambered no. 1.5 German Coverglass System (Nalge Nunc International, Naperville, IL, USA) at a density of 6.3 × 10^4^ cells/cm^2^. Seven days after seeding, the growth medium was replaced with liposomal suspension, and cells were incubated for 1 h at 37°C. Cellular uptake was terminated by washing three times with ice-cold PBS and the cell monolayers were fixed with 0.5 mL of 4% paraformaldehyde. The fixed cells were observed using an LSM 700 CLSM (Carl Zeiss, Gottingen, Germany).

### 2.3. Cytotoxicity Assay

Caco-2 cells were seeded in 96-well plates at a density of 3.15 × 10^4^ cells/cm^2^. Cytotoxicity was assessed using the CellTiter 96 AQueous One solution assay (Promega). The solution reagent contained MTS and an electron-coupling reagent (phenazine ethosulfate). Seven days after seeding, various concentrations of liposomes were added to the wells. The cultures were further incubated for 2 h, and then 20 *μ*L AQueous One Solution reagent was directly added to the culture wells. After 2 h of incubation, the absorbance of the produced color was measured with a microplate reader (MTB 120, Corona Electric, Japan) at a wavelength of 490 nm. The quantity of the formazan product, as measured by the absorbance at 490 nm, was directly proportional to the number of living cells in the culture.

### 2.4. *In Vivo* Animal Studies

Male Wistar rats (10-11 weeks old, SLC, Japan) were used in all *in vivo* studies. All experiments were approved and monitored by the Institutional Animal Care and Use Committee of Gifu Pharmaceutical University, and experiments were performed in line with Japanese legislation on animal studies. Before the experiments, the rats were fasted for 24 h with free access to water. The mucosal association and penetration of QD-loaded liposomes were visualized by CLSM using a method described previously [17]. The test samples were orally administered to the rats using intragastric tubes. The administered samples had a lipid concentration of 10 mM. The rats were sacrificed at 2 h after oral administration. The freshly excised tissues were fixed in Tissue-Tek Compound by immersion into liquid nitrogen. The molded samples were sectioned (10 *μ*m) using a cryomicrotome (Leica CM, Germany) and imaged by CLSM.

## 3. Results and Discussion

### 3.1. Characterization of Hydrophobic (Unmodified) QD-Loaded Liposomes

**Figure 1:**
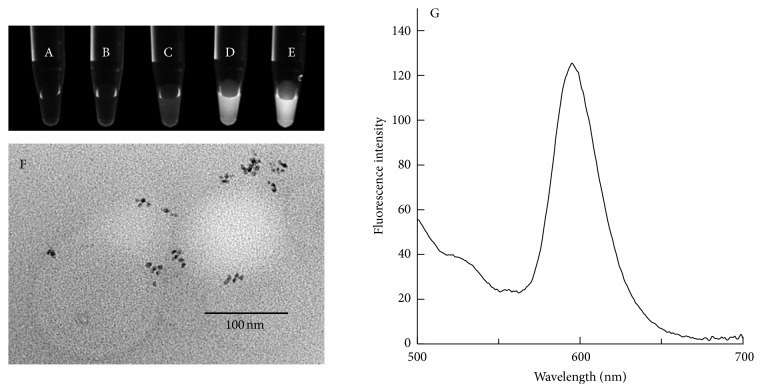
Physicochemical properties of hydrophobic (unmodified) QD-loaded SA liposomes. The lipid composition was DSPC : SA : Chol (= 8 : 0.2 : 1). Photographs of QD-loaded liposomes in phosphate buffer, (A) liposomes without QD before ultracentrifugation, (B) liposomes without QD after ultracentrifugation, and (C) 100 nM, (D) 200 nM, and (E) 400 nM QD-loaded liposomes after ultracentrifugation. All photographs were captured by placing the samples under a UV lamp. (F) TEM image of QD-loaded liposomes. The scale bar is 100 nm. (G) The fluorescence spectrum of QD-loaded liposomes was measured in phosphate buffer. Excitation was performed at 480 nm.

The average particle size of CdSe/CdZnS QDs with a core-shell structure was approximately 6.8 nm as measured by DLS. The fluorescence spectrum of CdSe/CdZnS QDs in chloroform was measured at an excitation wavelength of 480 nm. Emission was observed at 600 nm, and the spectrum had a symmetrical shape. In general, hydrophobic materials are incorporated into the liposomal lipid bilayer [18]. Therefore, we attempted to embed hydrophobic (unmodified) QDs into the liposomal lipid bilayer and examine the effects of QD concentration on liposomal properties. The particle size of QD-loaded liposomes was approximately 100 nm irrespective of the QD concentration (0–400 nM). These liposomes exhibited a positive zeta potential (approximately 20 mV), derived from the SA amino acid group. To confirm that QD fluorescence is preserved after incorporation into the lipid bilayer and that the liposomal bilayers were fluorescent, the liposomal preparations with different QD concentrations were placed under a UV lamp immediately after formation (Figures 1(A)–1(E)). The liposomes lacking QDs (negative control) emitted almost no fluorescence irrespective of ultracentrifugation (Figures 1(A) and 1(B)). QD-loaded liposomes in phosphate buffer exhibited fluorescent signals. Furthermore, the signals became stronger as the QD concentration increased (Figures 1(C)–1(E)). We considered that QDs were associated with the lipid bilayer via a hydrophobic interaction. No difference was observed in the pattern of the fluorescence spectrum of QDs after incorporation into the liposome compared with that of free QDs (Figure 1(G)), which suggested that QD degradation was not induced by the preparation process, and intact QDs were embedded in the lipid bilayers. To visualize how QDs are distributed inside the liposomal lipid bilayer, we collected and analyzed the images captured by TEM (Figure 1(F)). Some of the liposomes were still QD-free, but some QDs were loaded into the lipid bilayer. Most of the QDs were scattered along the lipid bilayer in a disorderly manner.

### 3.2. Characterization of GSH-QD-Loaded Liposomes

We selected GSH as a surface modifier for QDs to confer hydrophilicity to them. GSH is a tripeptide (*γ*-l-glutamyl-l-cysteinylglycine) and a biocompatible material that exists in most organs. GSH-QDs were prepared by a method reported previously [14]. In brief, the hydrophobic CdSe/CdZnS QDs surrounding trioctylphosphine oxide and hexadecylamine molecules are dispersed in tetrahydrofuran (THF). Then, a GSH solution is added to the QD solution in THF, and ligand exchange is performed at 60°C. After the deprotonation of the GSH carboxyl groups with potassium *t*-butoxide, GSH-coated CdSe/CdZnS QDs are easily dispersed in water. The particle size of GSH-QDs (6.5 nm) and their fluorescence properties in phosphate buffer were similar to those of unmodified QDs in chloroform (data not shown). GSH-QDs were encapsulated in liposomes via the hydration of lipid film using a GSH-QD suspension. Liposomes with different surface charges were examined; the negatively charged liposomal constitution was DSPC : DCP : Chol (8 : 2 : 1), and the positively charged constitution was DSPC : SA : Chol (8 : 0.2 : 1). 

**Figure 2:**
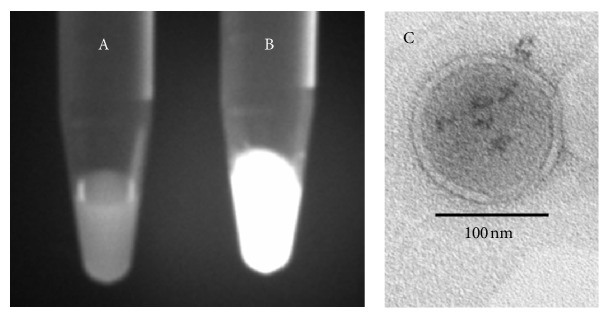
Physicochemical properties of GSH-QD-loaded liposomes with different surface charges. Photographs of GSH-QD-loaded liposomes in phosphate buffer: (A) DCP liposomes (DSPC : DCP : Chol = 8 : 2 : 1) and (B) SA liposomes (DSPC : SA : Chol = 8 : 0.2 : 1). All photographs were captured by placing the samples under a UV lamp. (C) Representative TEM image of a negatively stained GSH-QD-loaded SA liposome. The scale bar is 100 nm.

The fluorescence of GSH-QD-loaded liposomes was observed under a UV lamp for different liposomal compositions (Figures 2(A) and 2(B)). The GSH-QDs in the positively charged SA liposomes emitted strong fluorescence (Figure 2(B)). In contrast, the signal in the DCP liposome was less intense (Figure 2(A)). The GSH-QDs were negatively charged because of the dissociation of the carboxyl group of GSH. Therefore, the GSH-QDs could interact with SA liposomes electrostatically. These results suggested that the GSH-QDs were present in the inner aqueous phase of the liposome and that they were adsorbed onto the SA lipid bilayer electrostatically, whereas the GSH-QDs did not associate with the DCP lipid membrane of liposomes. As a result, GSH-QD-loaded SA liposomes displayed strong fluorescence compared with GSH-QD-loaded DCP liposomes. The DLS particle size of GSH-QD-loaded SA liposomes (219.5 nm) was larger than that of unloaded (115.4 nm) and GSH-QD-loaded DCP liposomes (104.7 nm) because of the interaction of QDs with SA on the liposomal surface. These considerations were supported by TEM images of GSH-QD-loaded SA liposomes (Figure 2(C)). GSH-QDs were observed in the aqueous core as well as on the SA liposomal surface.

### 3.3. *In Vitro* Detection of Liposomes in Caco-2 Cells after Exposure to QD-Loaded Liposomes

We have developed several types of liposomes modified with mucoadhesive polymers such as chitosan (positively charged polymer) or Carbopol (negatively charged polymer) as oral drug delivery systems. The liposome surface modification was achieved by simply mixing surface modifier solutions with negatively or positively charged (counter charged for surface modifier) core liposomes; electrostatic adsorption onto the charged liposomes was spontaneous [4]. Therefore, we evaluated the cellular association of DCP (negative) and SA (positive) liposomes. 

**Figure 3:**
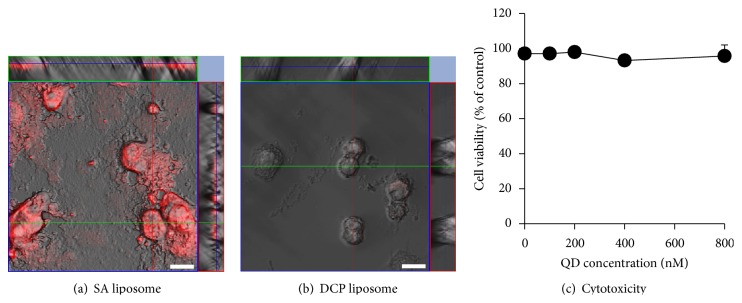
CLSM images and cytotoxicity study of Caco-2 cells following a 1h uptake of hydrophobic (unmodified) QD-loaded liposomal preparations: (a) SA liposomes (positive zeta potential) and (b) DCP liposomes (negative zeta potential). The QD concentration was 800 nM. Scale bars represent 20 *μ*m. (c) Cytotoxic side effects of QD-loaded SA liposomes at QD concentrations ranging from 0 to 800 nM on Caco-2 cells at 37°C. The liposomal exposure time of cells was 2 h. The viability of treated cells was determined by the MTS assay. Symbols represent the mean ± standard deviation (*n* = 6).

The cellular uptake of the different QD-loaded liposomal preparations was evaluated visually using CLSM (Figures 3(a) and 3(b)). CLSM revealed fluorescence activity in the Caco-2 cells exposed to QD-loaded liposomes. In *in vitro* cytotoxicity tests (Figure 3(c)), QD-labeled liposomes did not negatively affect the viability of Caco-2 cells during the uptake experiments. The images shown are z-sections through the center of the cells, which indicated that the fluorescence observed was the result of liposomal localization inside the cells. The uptake fluorescence of DCP liposomes with a negative zeta potential and SA liposomes with a positive zeta potential could be observed in the cytosol after analyzing the Caco-2 cells by CLSM, suggesting that liposomes were internalized by the Caco-2 cells. The uptake of SA liposomes into the Caco-2 cells was higher than that of DCP liposomes. The zeta potential of SA liposomes was positive (approximately 20 mV). These results suggested that cationic SA on the surface of liposomes enhanced the association between SA liposomes and negatively charged cell membranes via electrostatic interactions and that this association might serve to increase cellular uptake. 

### 3.4. *In Vitro* Cytotoxicity Study Using Caco-2 Cells

The potential toxic effects of QD-loaded liposomes were evaluated *in vitro* using Caco-2 cells by the MTS assay, which is a well-established technique for assessing toxicity (Figure 3(c)). The viability of Caco-2 cells was almost unchanged with exposure to QD-loaded liposomal suspensions in the range used in the cell studies that followed. This confirmed the very low cytotoxicity of these QD-loaded liposomes. 

### 3.5. *In Vivo* Detection of QD-Loaded Liposomes in the Intestinal Region after Oral Administration

**Figure 4:**
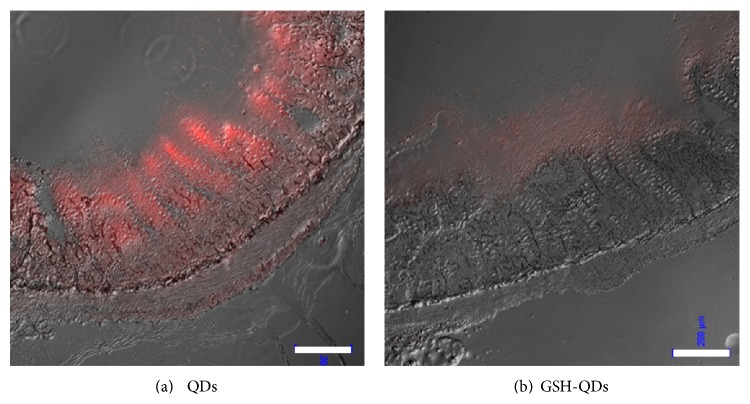
CLSM images of cryosections (ileum) after 2 h of oral administration of the different QD-loaded SA liposomes: (a) QD-loaded liposome and (b) GSH-QD-loaded liposome. The QD concentration was 800 nM. Scale bars represent 200 *μ*m.

The specific adhesion and uptake of QD-loaded liposomes into the intestinal mucosa were evaluated 2 h after oral administration.  Figure 4 shows the fluorescence signals in the mucosal tissues of intestinal specimens after oral administration of liposomes. The intestinal tubes were removed from the rats at the appropriate time after oral administration of the liposomes. The ileum was sliced for CLSM observation. The detectability of QD and GSH-QD-loaded liposomes in the intestinal tube was evaluated by observing the residual liposomes on the mucosa. Small amounts of GSH-QDs were observed (Figure 4(b)), whereas large amounts of hydrophobic QDs were detected by CLSM (Figure 4(a)). These results suggested that unmodified QD-loaded liposomes were better for tracing and detecting liposomes in the body after oral administration. 

We confirmed that the fluorescence activity of GSH-QDs disappeared in an acidic pH buffer that mimicked gastric juice (data not shown). The dissociation of GSH from the QD surface and degradation of QDs could be induced by gastric juice in the stomach [19]. In contrast, QDs embedded in the lipid bilayers were protected by the surrounding lipids from the acidic condition of the gut (Figure 4(a)). Therefore, GSH-QDs were not suitable as labeling markers of orally administered liposomes. However, this mucoadhesive liposome system was investigated for other administration routes such as pulmonary and eye-drop administration [20, 21]. GSH-QDs entrapped in liposomes appear to be effective for analyzing liposomal behavior in the body for administration routes that do not involve the stomach.

Hydrophobic (unmodified) QDs were incorporated into the lipid bilayer. Conversely, there were GSH-QDs in the inner aqueous phase. Therefore, the combination of QDs and GSH-QDs might be effective for liposomal detection.

In the present study, non-NIR QD-loaded liposomes were orally administered to rats. The main purpose of this study was to evaluate whether orally administered QDs could be used as fluorescence markers as a replacement for organic fluorescence materials. We consider this experiment useful for illustrating the tracing potential of QDs for orally administered liposomes. Further investigation is required to optimize the efficacy of NIR-QDs in noninvasive imaging for evaluating the pharmacodynamics of liposomes and the mucoadhesive effects of surface modifiers.

## 4. Conclusions

In summary, we prepared two types of QD-loaded liposomes: unmodified (hydrophobic) QDs embedded in the lipid bilayer and GSH-QDs dispersed in the inner aqueous phase of liposomes. QD-loaded liposomes had high biocompatibility and low toxicity in Caco-2 cells. Using CLSM, the fluorescent signal of QDs in the liposomes could be detected in the intestinal mucosa after oral administration. These results suggested that QDs are useful materials as tracers of liposomes in *in vivo* applications.
